# Supplementary material for: Calcium signals are necessary to establish auxin transporter polarity in a plant stem cell niche
Source: Nat Commun. 2019 Feb 13;10:726. doi: 10.1038/s41467-019-08575-6 (PMC6374474; doi:10.1038/s41467-019-08575-6)
Supplement: Supplementary file 1 — Supplementary Information [file 41467_2019_8575_MOESM1_ESM.pdf]

Supplementary Information

Calcium signals are necessary to establish auxin transporter polarity in a plant stem cell niche

Li, T., Yan, A., Bhatia, N. *et al.*

Supplementary Figures

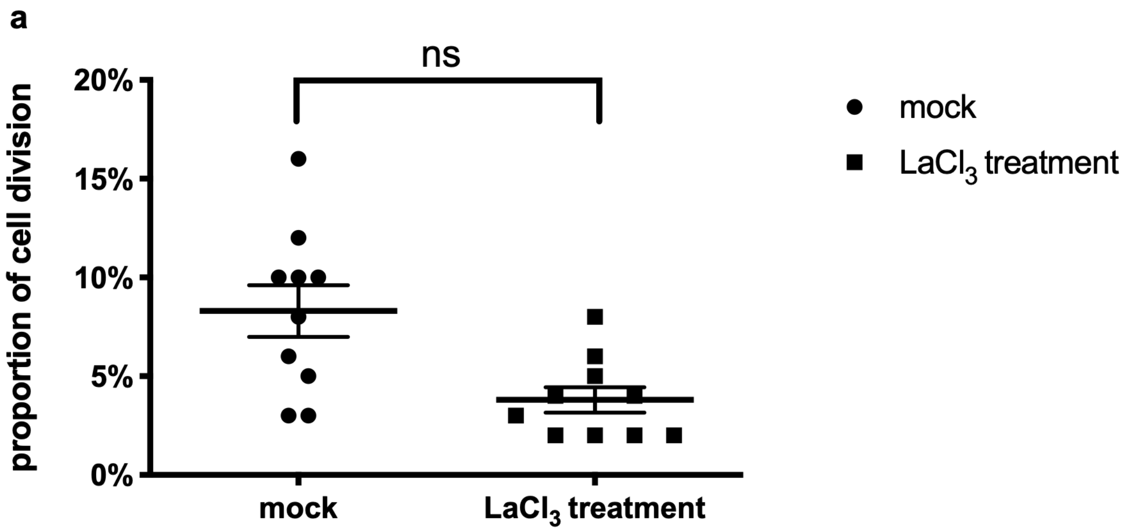

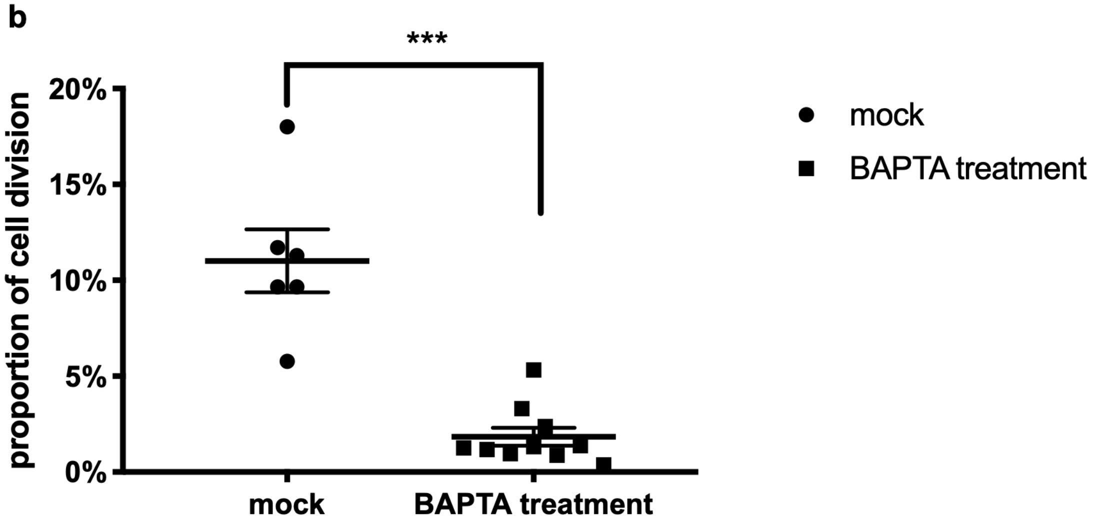

Supplementary Figure 1. Cell division rates of the samples during 12h of growth under $LaCl_3$ and BAPTA treatments. a, Proportion of epidermal cells dividing in SAMs under mock treatment and with 5mM $LaCl_3$ repeatedly applied for 2 min each hour (without a subsequent water rinse) for 12h. Images were captured at 0h and 12h. The proportion was calculated as the number of cells that divided over 12 h compared to the total cell number in the SAM region (a

region that did not include flower primordia). n = 10 for both mock treatment and $LaCl_3$ treatment. P value = 0.1641 by Kolmogorov-Smirnov test. **b,** Proportion of epidermal cells dividing with and without BAPTA treatment. Treatment was for 2 min each hour, without subsequent washout, for a total of 12 h. n = 6 for both mock treatment and n = 10 for BAPTA treatment. *** indicates P value < 0.001. P value = 0.0002 by Kolmogorov-Smirnov test. Lines are at mean with standard error of the mean. Source data for a and b are provided as a Source Data file.

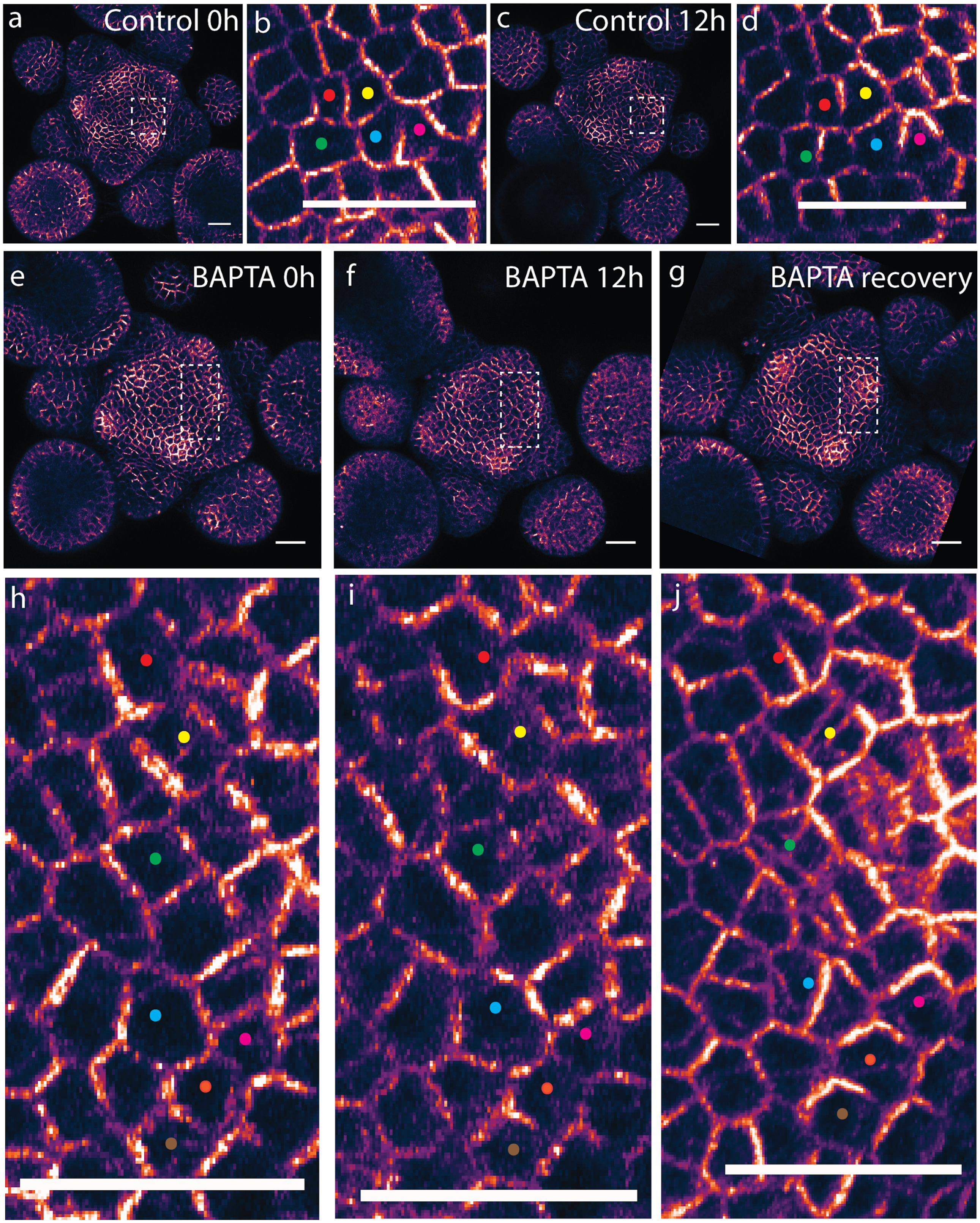

Supplementary Figure 2. PIN1 dynamic polarization pattern during 12h growth after

hourly 2mM BAPTA treatment. a, c, PIN1-GFP signal indicates a new primordium forming after 12 h (dashed square). **b**, **d**, Enlargement of the square region in (**a**, **c**). Colored dots indicate the cells in which PIN1-GFP polarity changed over 12h of growth. **e-g**, The usual dynamic pattern of PIN1-GFP polarization becomes static after repeated application of 2mM BAPTA for 2 min in every hour for 12 h without a following sample rinse, region enlarged in **h-j** indicated by a dashed rectangle (**f**), but primordia resume growth and PIN1-GFP signal re-localizes in the rectangular region after 12 h of recovery initiated by a 3mM $CaCl_2$ rinse (**g**). **h**-**j**, Enlargement of the rectangular region in (**e**-**g**). The cells that are labeled by colored dots show that PIN1-GFP do not have polarity changes during BAPTA treatment but resume the dynamic pattern after 12 h recovery. **a**-**j**, scale bar: 20 μm. LUT is gem in Fiji-ImageJ software.

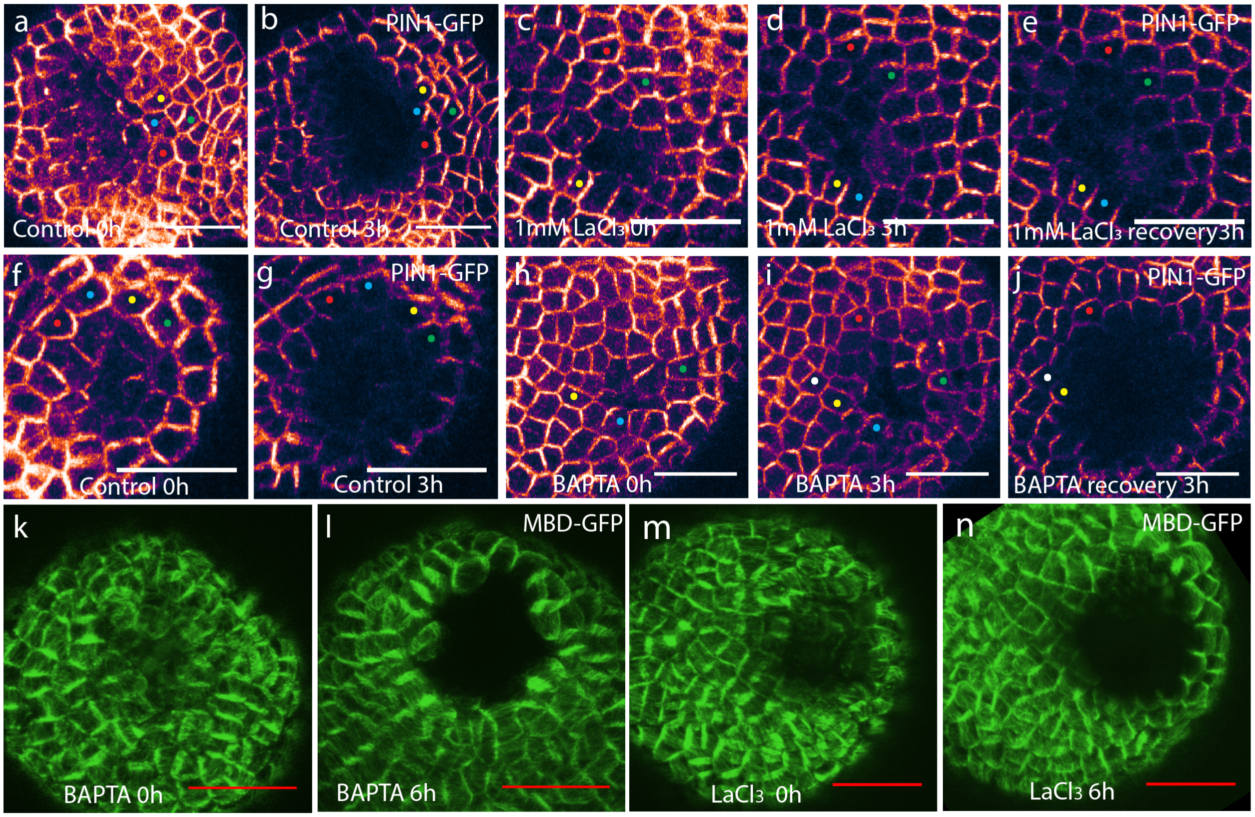

Supplementary Figure 3. PIN1 and MT mechanical responses after pretreatment with BAPTA and $LaCl_3$. a, **b**, PIN1-GFP shows polarized subcellular localization 3h after local cell ablation with a glass pipette, without pharmacological treatment. **c**, **d**, PIN1-GFP reorientation was partially detected 3h after cell ablation following 15 min pretreatment with 1mM $LaCl_3$ without a subsequent water rinse. **e**, At 3 additional hours after $LaCl_3$ washout, PIN1-GFP has continuously reoriented away from the ablation site. **f**, **g**, Control experiments show PIN1-GFP polarization pattern 3h after cell ablation. Signal was color-coded. **h**-**j**, Impact of a 10 min, 2mM BAPTA pretreatment on PIN1 behavior at 0 h (**h**) and 3 h (**i**) after cell ablation without a subsequent sample rinse, and PIN1 mechanical response recovery 3 h after BAPTA washout and $CaCl_2$ (3mM) resupply (**j**). **a-j**, LUT is gem in Fiji-ImageJ software. Similarly colored dots mark the same cells tracked over time. **k-n**, Z-stack maximum projection showing that MBD-GFP

reorients circumferentially around ablated cells 6 h after perturbation in a SAM after a 10 min pretreatment with 2mM BAPTA (**k**, **l**) and 15 min pretreatment with 5mM $LaCl_3$ (**m**, **n**). **a**-**n**, Scale bar: 20 µm.

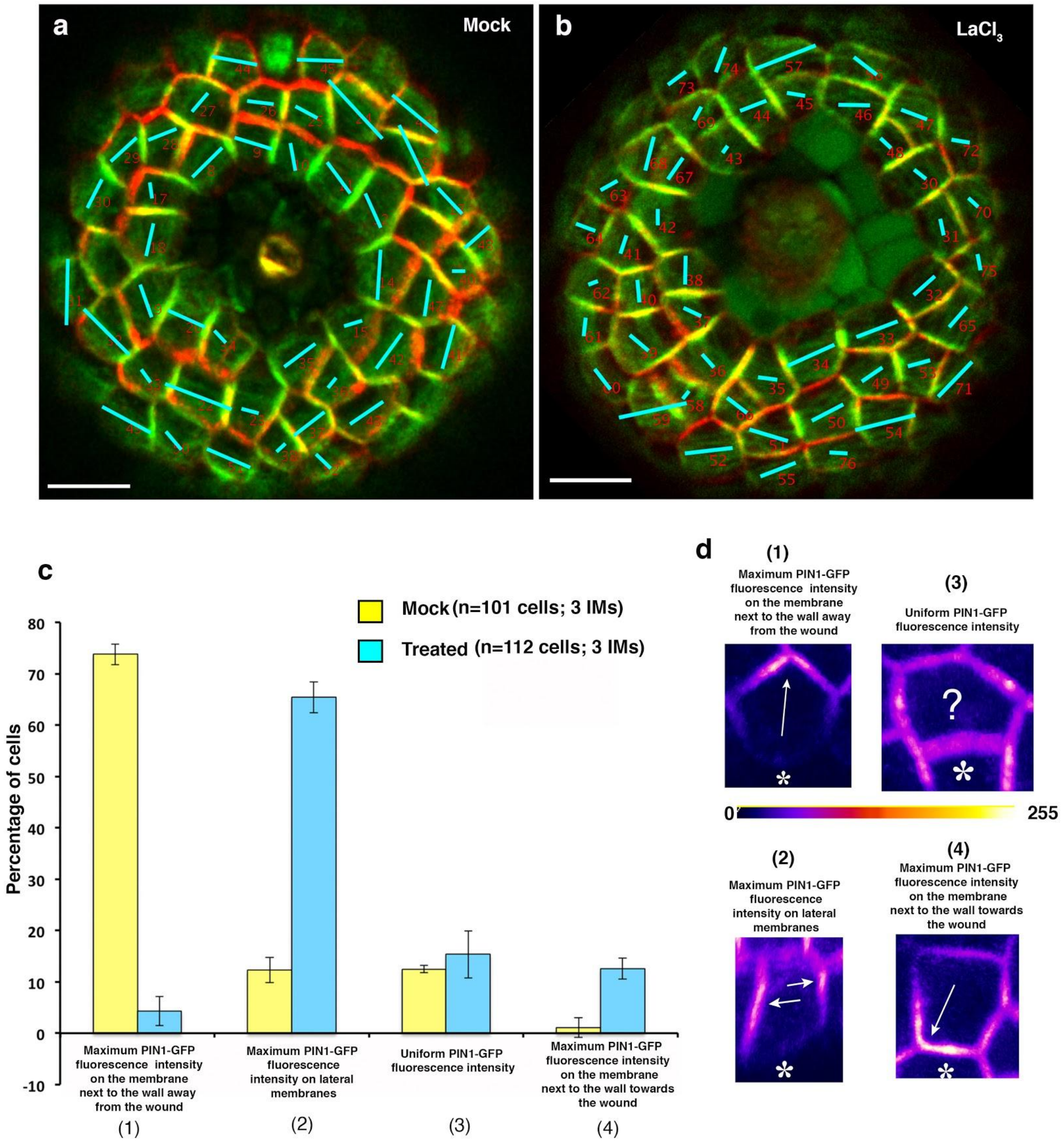

Supplementary Figure 4. Quantification of cortical MT orientation and estimation of maximum PIN1-GFP fluorescence localization in response to laser-induced mechanical perturbation following a 15 min pretreatment with 5mM $LaCl_3$

a and **b**, Close up views of the inflorescence meristems pretreated with mock solution (**a**) and 5mM $LaCl_3$. **b,** expressing *pML1::mCherry-MAP4* (green) and *pPIN1::PIN1-GFP* (red), showing calculated cortical MT orientations (blue lines) 4 hours after laser induced cellular

ablations using FibrilTool. The angle of the blue lines represents average orientation and their length is proportional to the MT array anisotropy[1]**.** Blue lines were manually drawn to overlay the original lines from FibrilTool for better visualization. **c**, Estimation of PIN1-GFP signal localization upon cellular ablation in mock (n=101 cells; 3IMs) and $LaCl_3$ pretreated (n=112 cells; 3IMs) inflorescence meristems. The bar plot shows the percentage of cells with maximum PIN1-GFP fluorescence signal localisation divided into 4 categories- (1) maximum PIN1-GFP fluorescence intensity on the membrane on the side of the cells farthest from the site of ablation, (2) maximum PIN1-GFP fluorescence intensity on the membranes radially oriented with respect to the wound, (3) uniform PIN1-GFP fluorescence intensity and (4) maximum PIN1-GFP fluorescence intensity on the membrane adjacent to the site of ablation. Note, a much higher percentage of cells with maximum PIN1-GFP fluorescence intensity on the membranes furthest away from the site of ablation in mock treated meristems compared to the pretreated ones with $LaCl_3$. Source data are provided as a Source Data file. **d**, Representative images showing the four categories of maximum PIN1-GFP fluorescence signal localization described in (**c**). Asterisks in (**d**) indicates the direction of the cellular ablation site. Scale bar- 10 µm (**a** and **b**).

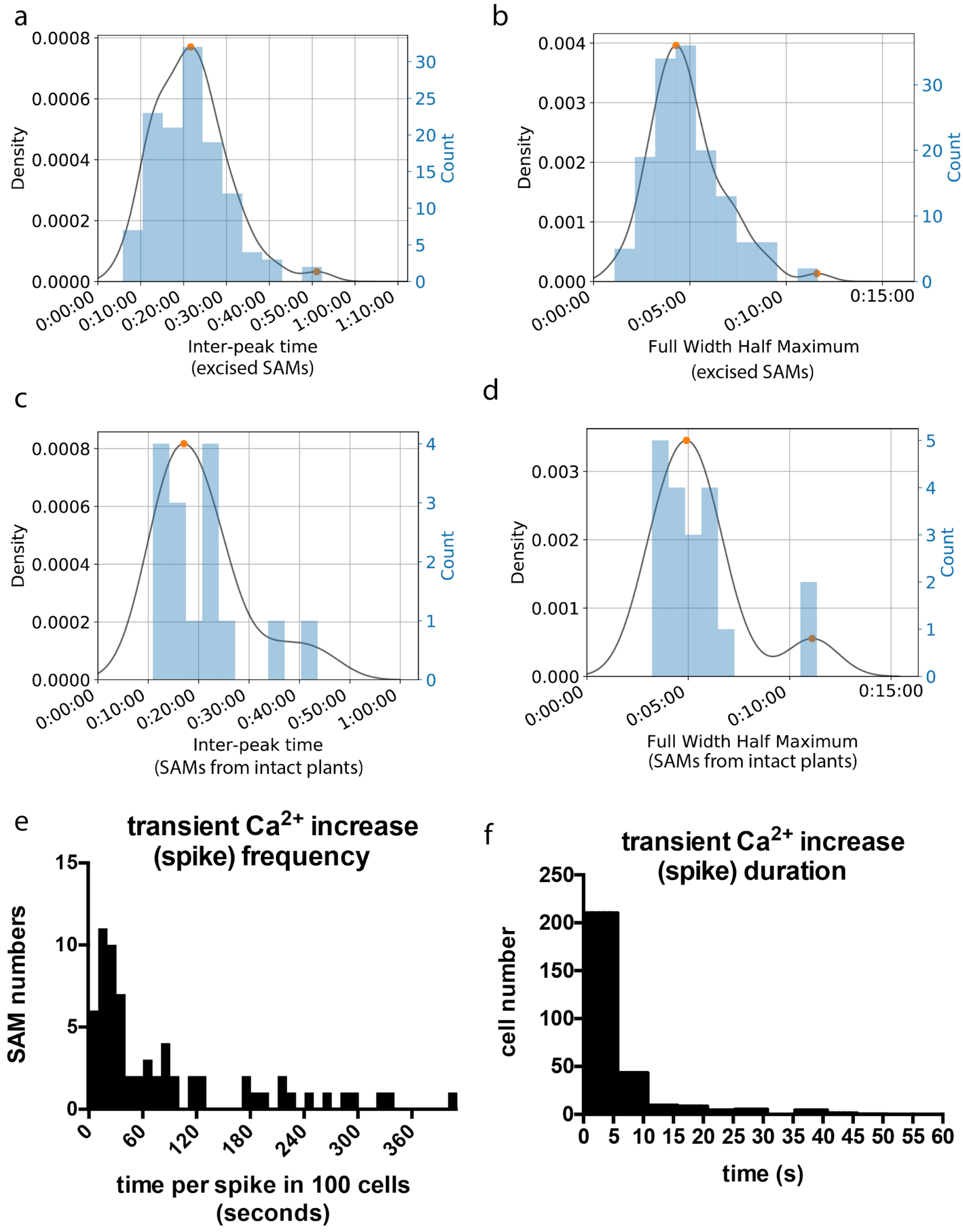

Supplementary Figure 5. Quantitative analysis of two different spontaneous Ca^{2+} signals. a, Statistics of the meristematic Ca^{2+} oscillation inter-peak times from excised SAMs: the panel

presents the histogram (blue bars, counts on right axis) and Kernel Density Estimation (KDE; gray curve, values on the left axis), resulting in a mode (orange dot) found at 21m40s. The sample median is 21m10s and the estimated mean ± relative standard error are 21m45s ± 4% (*n* = 123, SD = 8m35s). **b**, Statistics of the meristematic Ca^{2+} oscillation FWHM times from excised SAMs. The panel presents the histogram (blue bars, counts on right axis) and rectangles Kernel Density Estimation (KDE; gray curve, values on the left axis), resulting in a mode (orange dot) found at 4m17s. The sample median is 4m33s, and the estimated mean ± relative standard error are 4m51s ± 3% (*n* = 141, SD = 1m53s). **c,** Statistics of the meristematic Ca^{2+} oscillation inter-peak times from intact SAMs: the panel presents the histogram (blue bars, counts on right axis) and Kernel Density Estimation (KDE; gray curve, values on the left axis), resulting in a mode (orange dot) found at 17m05s. The sample median is 18m45s and the estimated mean ± relative standard error are 20m34s ± 11% (*n* = 15, SD = 8m55s). **d,** Statistics of the meristematic Ca^{2+} oscillation inter-peak times from intact SAMs: the panel presents the histogram (blue bars, counts on right axis) and Kernel Density Estimation (KDE; gray curve, values on the left axis), resulting in a mode (orange dot) found at 4m55s. The sample median is 5m17s and the estimated mean ± relative standard error are 5m32s ± 9% (*n* = 19, SD = 2m15s). **e**, Quantitative calculation of the spike signal frequency. The results are shown as the mean time that is necessary for one spike signal event among estimated area of 100 cells. *n* = 69 SAMs in the total experiments. **f**, Analysis of individual cell spike duration. n = 292 individual cells from 30 SAMs. Source data for a-f are provided as a Source Data file.

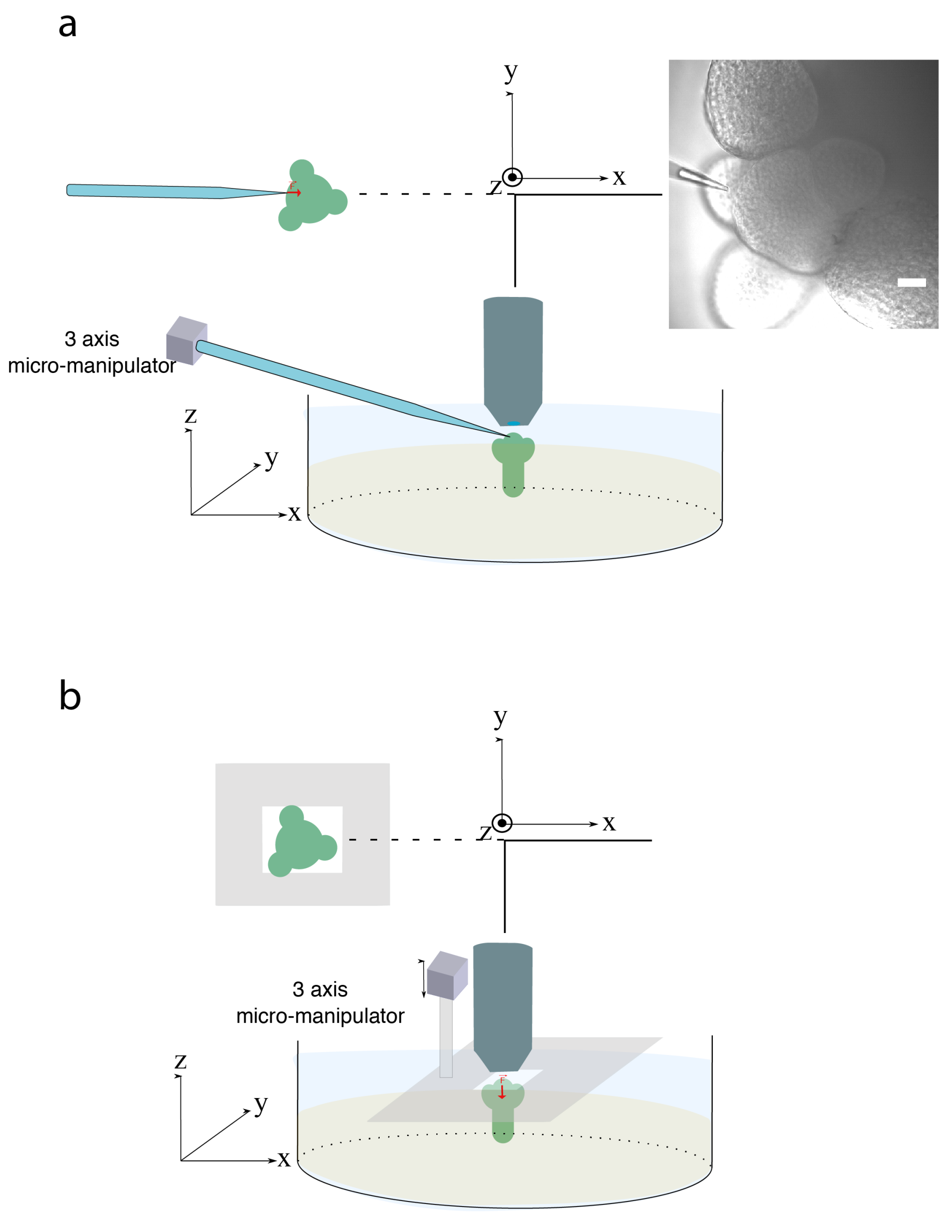

Supplementary Figure 6. Mechanical perturbation device configurations. a, Set-up configuration of the micropipette device used to apply and withdraw a glass pipette from the SAM. The upper-right picture represents a bright-field image of a SAM with a glass micropipette at its side. **b**, Set-up configuration of the compression device used to press on and release pressure from the top of the SAMs.

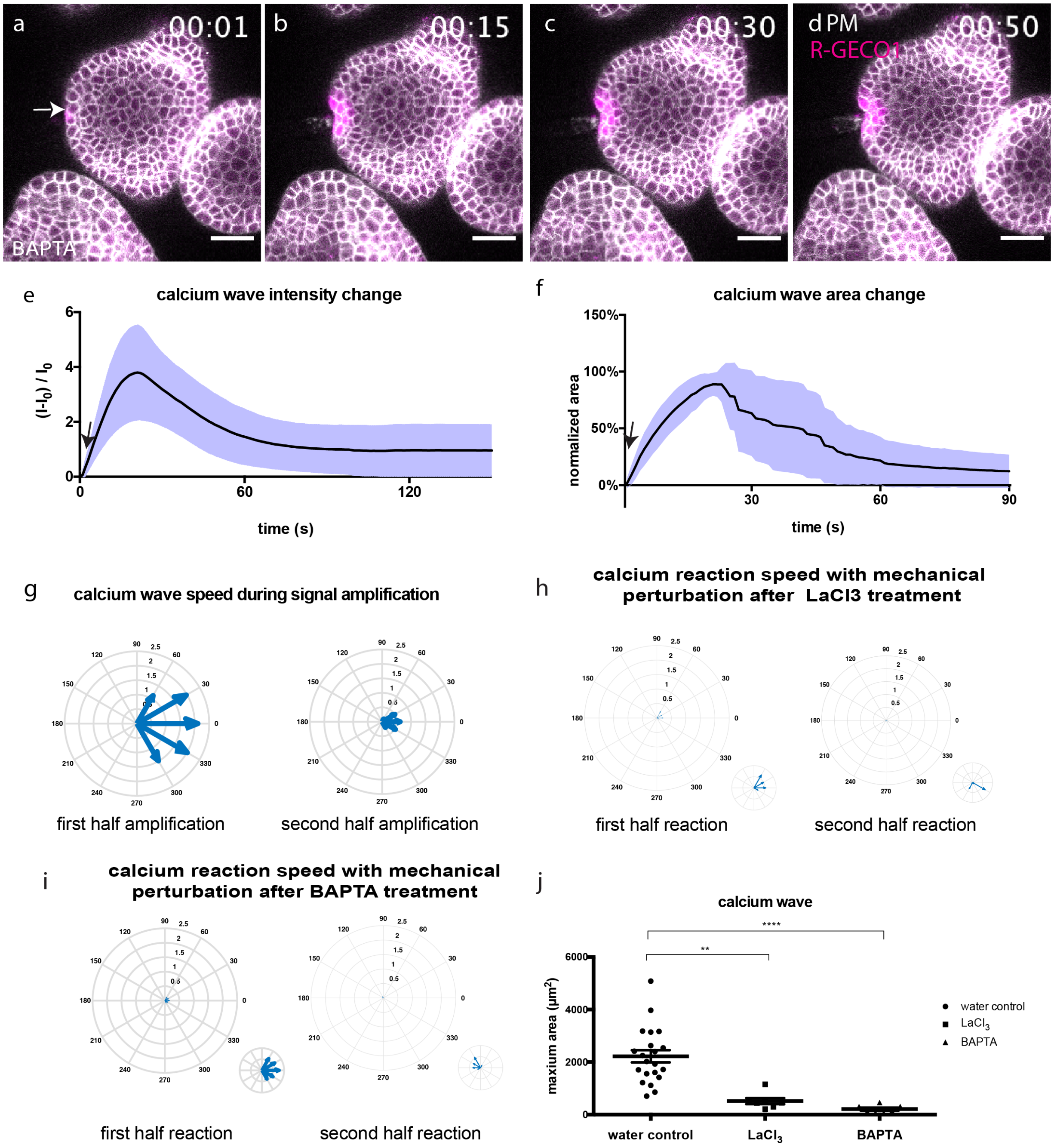

Supplementary Figure 7. Mechanical perturbation activated calcium waves are abolished after 15 min pretreatment with 5mM $LaCl_3$ or 10 min pretreatment with 2mM BAPTA. a-d, Representative frame images of R-GECO1 (magenta) and membrane marker 29-1 fused to GFP (gray) in the SAM to show effects of 10 min pretreatment with 2mM BAPTA on Ca^{2+} wave transmission in the SAM. Arrow points to the direction of pipette prodding. Scale bars, 20 μm

(**a-d**). Time format mm:ss (**a-d**). **e**, Quantitative analysis of Ca^{2+} signal in the SAM in response to mechanical stimulus. $(I-I_0)/I_0$ shows mean normalized R-GECO1 fluorescence intensity fold change. Purple represents SD of 26 independent experiments. **f**, Quantitative calculation of normalized area of the Ca^{2+} signal propagation region. Purple represents SD of 20 independent experiments from 12 SAMs. Arrows in (**e**,**f**) point to the time stimulation started. **g**, Quantitative measurement of Ca^{2+} wave propagation speed in five different directions (-60°, -30°, 0°, 30°, 60°), during signal amplification. unit: µm/s. Left panel shows the information in first half propagation (by time) during signal amplification, right panel shows the information in second half propagation (by time). **h**, Quantitative measurement of Ca^{2+} reaction propagation speed in different directions after 5mM $LaCl_3$ pretreatment. unit: µm/s. Small panel in the bottom-right corner shows the speeds at larger scale. Left panel shows the information in the first half time of the wave propagation, right panel shows the information in the second half time of the wave propagation. **i**, Quantitative measurement of Ca^{2+} wave propagation speed in different directions after 2mM BAPTA pretreatment. unit: µm/s. Small panel in the bottom-right corner shows the speeds at larger scale. Left panel shows the information in first half time of the propagation, right panel shows the information in the second half. **j**, Area of calcium waves at maximum spread after mechanical perturbation during water control, $LaCl_3$ and BAPTA treatments. Each symbol represents one SAM. Horizontal bars represent mean ± SEM. ** indicates P value < 0.01 (P value = 0.0034 by Kruskal-Wallis with Dunn's multiple comparison test), **** indicates P value < 0.0001 (P value is calculated by Kruskal-Wallis with Dunn's multiple comparison test). Source data for e-j are provided as a Source Data file.

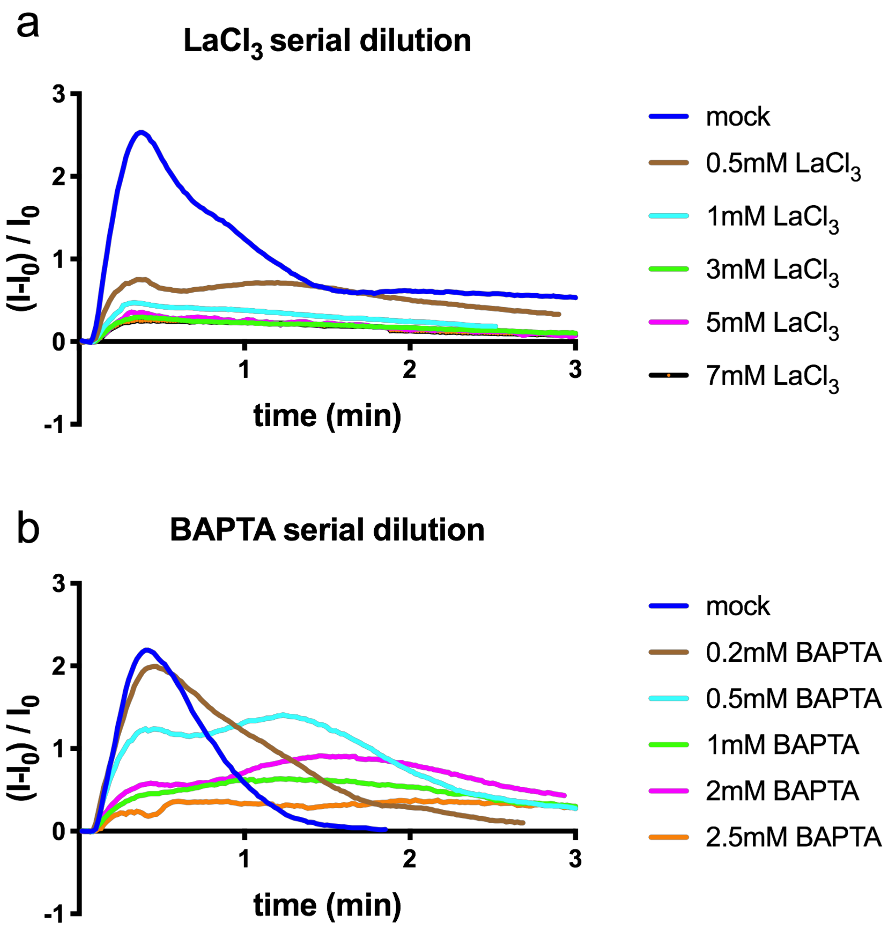

Supplementary Figure 8. Effect of serial dilution of $LaCl_3$ and BAPTA on mechanically induced Ca^{2+} signals. a, R-GECO1 fluorescent signal intensity fold change $(I-I_0)/I_0$ after a 15 min SAM pretreatment with different concentrations of $LaCl_3$ followed by mechanical ablation with a glass micropipette. *n* = 6, 9, 6, 5, 7, 4 for mock, 0.5, 1, 3, 5, 7mM $LaCl_3$ treatments respectively. **b,** R-GECO1 fluorescent signal intensity fold change $(I-I_0)/I_0$ from a 10 min SAM pretreatment with different concentrations of BAPTA followed by mechanical ablation with a glass micropipette. *n* = 5, 7, 5, 5, 8, 4 for mock, 0.2, 0.5, 1, 2, 2.5mM BAPTA treatments respectively. **a** and **b**, A 2D plane of the meristem region was defined as the region of interest. Lines represent mean of fold changes. Source data for a and b are provided as a Source Data file.

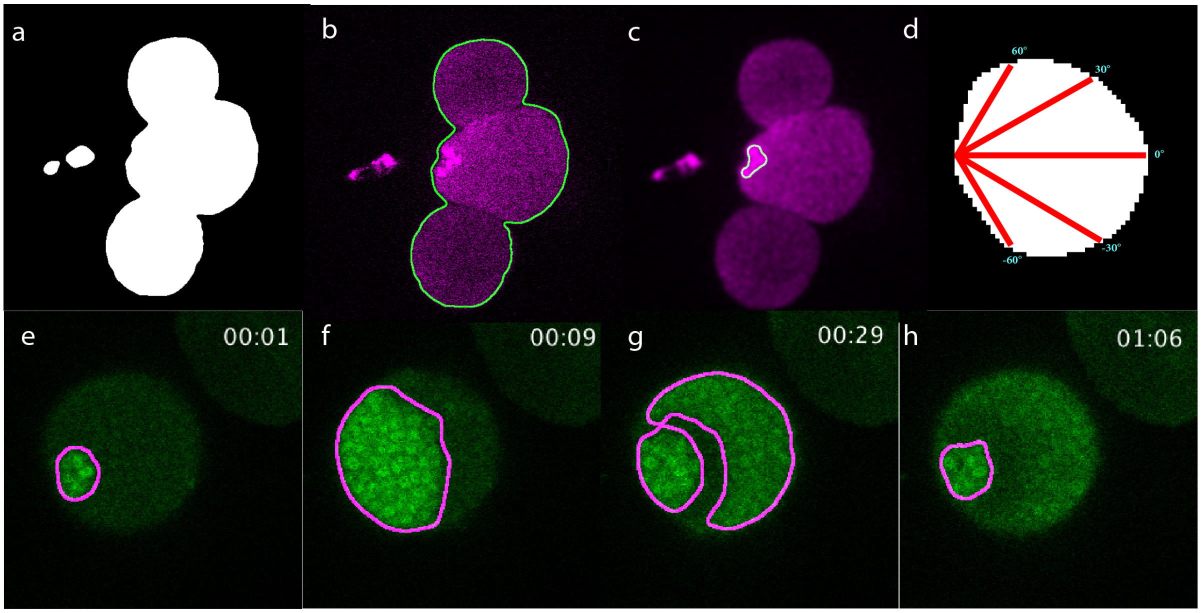

Supplementary Figure 9. Image analysis and speed measurements of the Ca^{2+} wave. a, The mask of the original frame of the SAM tissue. Possible artifacts that are outside of the mask were removed from the mask by keeping only the largest area representing the tissue. The overlay of the tissue boundaries is shown in (**b**). The final segmented region shows the Ca^{2+} signal as overlaid onto the original frame in (**c**). (**d**) shows a segmented signal region with lines linking the mechanical stimulation site to the boundary pixels in five different directions for distance measurements. **e-h**, Representative frame images show Ca^{2+} signal segmentation during wave propagation after mechanical stimulation with a glass micropipette.

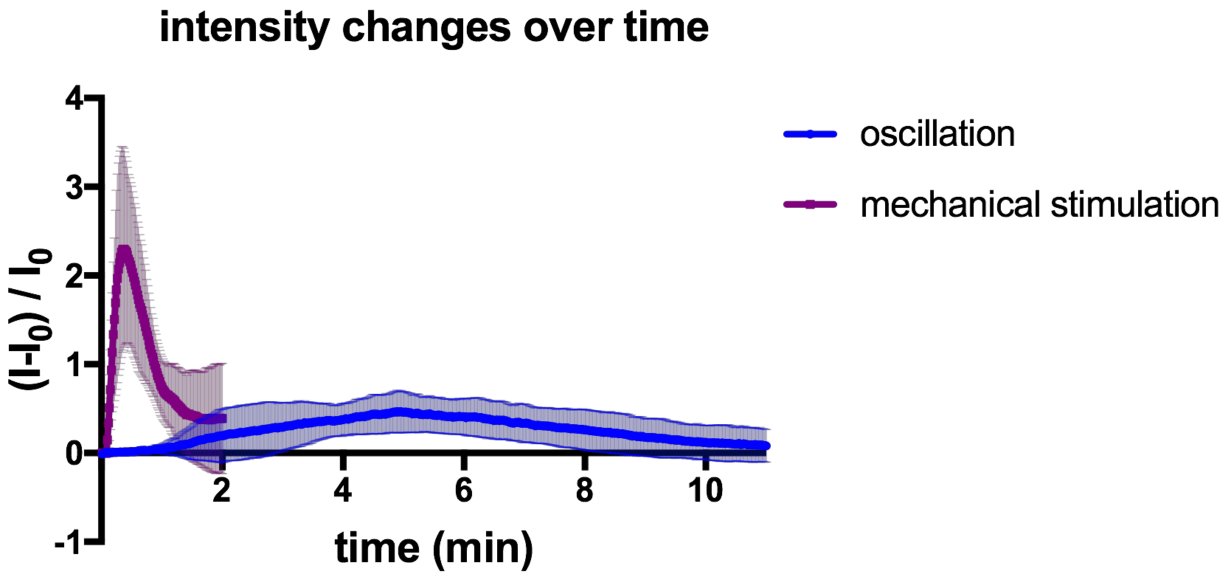

Supplementary Figure 10. Quantitative comparison of the fluorescence intensity differences between spontaneous Ca^{2+} signal oscillations and Ca^{2+} waves in response to mechanical ablation by a glass pipette. Lines show the mean of normalized intensity fold changes. Error bars represent SD of 12 oscillation peaks (blue) and 7 wave peaks (purple). Source data are provided as a Source Data file.

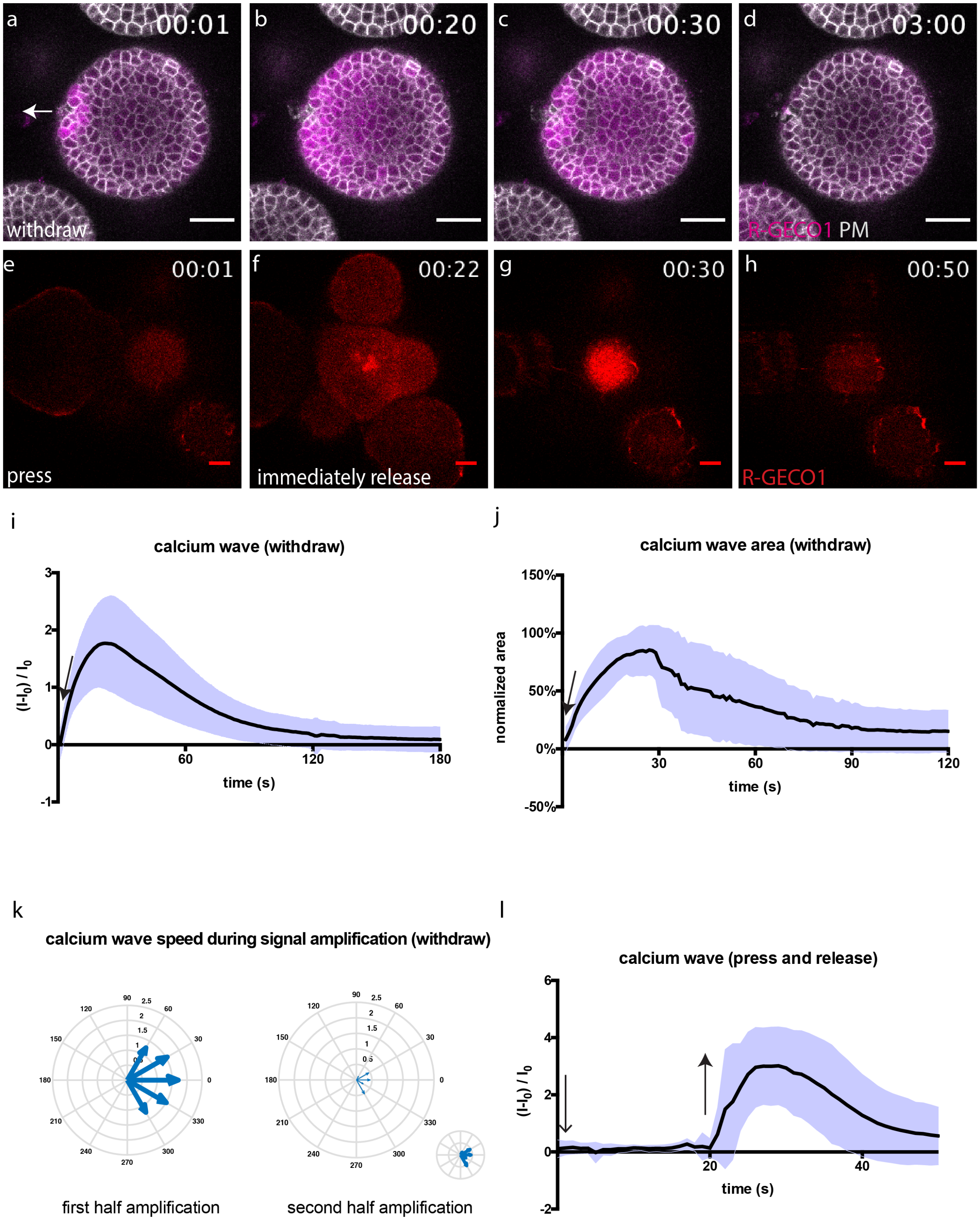

Supplementary Figure 11. Ca^{2+} signal responses to non-injurious mechanical perturbations. a-d, Representative frame images of R-GECO1 (magenta) and membrane marker

29-1 fused to GFP (gray) in the SAM, in response to stimulus from a glass pipette when withdrawing from the SAM. Arrow points to the direction of pipette withdrawal. **e**-**h**, Representative frame images of R-GECO1 in the SAM, in response to mechanical stimulus from a cover slip when pressing and releasing from the top of the SAM. Resting Ca^{2+} signal before mechanical perturbation as shown in (**e**). **f**, R-GECO1 signal does not change dramatically after applying the pressing force. **g**, R-GECO1 signal increases immediately after pressure is released, and recedes back to the resting level in approximately 15 seconds (**h**). **a**-**h**, Time format mm:ss. Scale bar: 20 µm. **i**, Quantitative analysis of Ca^{2+} signal in response to mechanical stimulus after withdrawing a glass pipette from the SAM. $(I-I_0)/I_0$ shows mean from normalized R-GECO1 fluorescence intensity-fold changes. Purple represents SD of 24 independent experiments from 20 SAMs. Arrow points to the time the pipette started to withdraw. **j**, Quantitative calculation of normalized Ca^{2+} signal propagation area after withdrawing a glass pipette from the SAM. Error bars represent SD of 15 independent experiments from 13 SAMs. Arrow points to the time the pipette started to withdraw. **k**, Quantitative measurement of Ca^{2+} wave propagation speed in different directions during signal amplification after the pipette is withdrawn. unit: µm/s. Left panel shows the information for the first half time of the wave propagation, right panel the second half time. Small panel in right corner shows the speeds at larger scale. $n = 21$ from 16 SAMs. **l**, Quantitative analysis of Ca^{2+} signal in response to mechanical stimulus when cover slip is pressed and released from the top of the SAM. The cover slip is lifted at around 20 seconds. $(I-I_0)/I_0$ shows mean from normalized R-GECO1 fluorescence intensity-fold changes. Purple represents SD of 6 independent experiments from 3 SAMs. Arrows represent the initial time of pressure application and then of subsequent release of the cover slip. Source data for i-l are provided as a Source Data file.

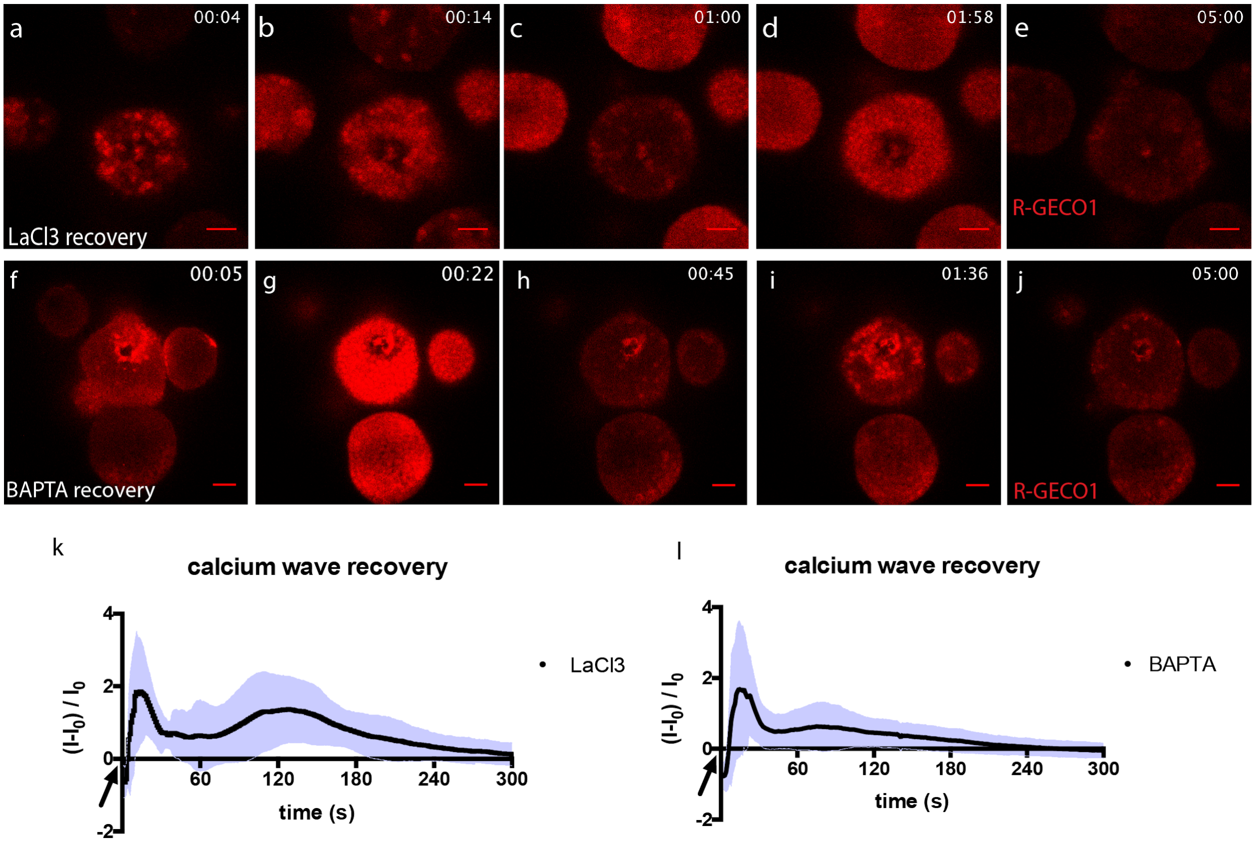

Supplementary Figure 12. Restoration of the Ca^{2+} wave at 3 h after mechanical stimulation with a pipette to SAMs pretreated with Ca^{2+} inhibitors. a-e, SAMs were pretreated with 5mM $LaCl_3$ for 15 min before mechanical stimulus and then subjected for 5 min to 5mM $LaCl_3$, which in the continued presence of inhibitors did not cause a calcium wave. After 3 h of incubation on GM without a sample rinse, the SAM was washed with water. A Ca^{2+} wave occurs immediately after the water wash. **f-j**, After 10 min pretreatment with 2mM BAPTA following mechanical stimulus and then subjected to 5 min of 2mM BAPTA, after 3h incubation without sample rinse, the Ca^{2+} wave was also restored following washout with 3mM $CaCl_2$. n = 9 of 19 independent experiments. **a-j**, Time format mm:ss. Scale bar: 20 µm. **k**, Quantitative analysis of induced Ca^{2+} signal recovery 3 h after $LaCl_3$ pretreatment, and immediately after water washout. $(I-I_0)/I_0$

shows mean of normalized R-GECO1 fluorescence intensity fold changes. Purple represents SD of 11 independent experiments. Arrow represents water wash initiation. **l**, Quantitative analysis of Ca^{2+} signal recovery 3 h after 2mM BAPTA pretreatment, immediately after 3mM $CaCl_2$ resupply. $(I-I_0)/I_0$ shows mean of normalized R-GECO1 fluorescence intensity fold changes. Purple represents SD of 9 independent experiments. Arrow represents the time the water wash began. Source data for k-l are provided as a Source Data file.

Timeline		Prodding (n=34)	Withdraw (n=21)
signal begins to decrease at the time	mean (s)	23	28
	SD (s)	3	5
dark crescent signal lasted for	mean (s)	16	19
	SD (s)	5	7
entire Ca^{2+} response time	mean (s)	73	92
	SD (s)	18	16
primary peak amplitude	mean of intensity fold change	3.8	1.8
wave speed (first half)	$\mu m\ s^{-1}$	~2	~1.5
wave speed (second half)	$\mu m\ s^{-1}$	~0.5	~0.5

Supplementary Table 1: The response timeline of Ca^{2+} wave propagation by prodding cells and pipette withdraw from the SAM. SD: Standard Deviation. s: seconds. Source data are provided as a Source Data file.

Timeline		$LaCl_3$ pretreatment (n=11)	BAPTA pretreatment (n=10)
primary peak initiated at the time	mean (s)	8	8
	SD (s)	3	3
primary peak lasted for	mean (s)	23	21
	SD (s)	8	8
primary peak amplitude	mean intensity fold change	2	1.7
two peaks interval	mean (s)	60	41
	SD (s)	25	16
secondary peak lasted for	mean (s)	81	88
	SD (s)	12	15
secondary peak amplitude	mean intensity fold change	1.5	0.6

Supplementary Table 2: The timeline of Ca^{2+} response during signal recovery after Ca^{2+} signal inhibition. SD: Standard Deviation. s: seconds. Source data are provided as a Source Data file.

Primer names	Primer sequences
GCaMP6F	5’GGATCCatgggttctcatcatcat3’
GCaMP6R	5’GGATCCtcacttcgctgtcatcatttgtac3’

Supplementary Table 3: A primer list

Supplementary Methods

Plant materials and growth conditions.

The DNA for *GCaMP6f(fast)* was obtained from the lab of Dr. Francesca Peri (EMBL Heidelberg). A 1356 bp fragment was amplified using primer pair GCaMP6F, R (Supplementary Table 3) and cloned downstream of the *UBQ10* promoter (1.9kb) via *BamH1* into a BJ36 vector carrying *ocs* terminator (730bp). *5'pUBQ10::GCaMP6f-ocs3'* fragment was then cloned into *pMOA34* binary vector (hygromycin resistant in plants) via *Not1* and transformed into *Arabidopsis (ler* ecotype). The transgenic plants were identified based on hygromycin resistance on plates containing GM (Growth Medium, containing 1% sucrose, 1X Murashige and Skoog salts (Sigma M5524), MES 2-(MN-morpholino)- ethane sulfonic acid (Sigma M2933) brought to pH 7 with 1M KOH, plus 0.8 % Bacto Agar (Difco), 1% MS vitamins (Sigma M3900)) supplemented with Hygromycin.

Construction of *pML1::mCherry-MAP4* has been described previously[2]. The fragment was cloned into *pMOA34* binary vector and transformed into plants carrying *pPIN::PIN1-GFP* transgene in *ler* ecotype[3].

The *Arabidopsis* R-GECO1 line (*Col-0* ecotype) is described in Keinath et al. 2015[4]. PIN1-GFP, MBD-GFP reporter lines were previously described[3,5]. The *pUBQ10::29-1-GFP* plasma membrane marker line was prepared for this study. *Arabidopsis thaliana* plants were grown in a sunshine soil/vermiculite/perlite mixture under continuous light at 20 °C.

Image processing and data analysis.

Quantitative characterization of the Ca^{2+} oscillations (Fig. 3a) in the excised SAM and intact SAMs was performed as follows: for each sample, the mean pixel intensity over a region of interest in the SAM was evaluated ('SAM signal'); to account for non sample-related contributions to the signal, this procedure was repeated in the same frames, with the region-of-interest defined to exclude the sample region ('BG signal'), which was then subtracted from the SAM signal; to suppress experimental noise and sporadic cellular spikes, natural smoothing cubic splines [6] were applied to the resulting signal. De Boor's smooth factor, ranging from zero to one, was chosen automatically, where the 'improved Akaike information criterion' (AIC_C)[7] took the role of the usual generalized cross-validation; smooth factor of value 1 leads to no smoothing (interpolating spline), while approaching zero converges to linear least squares. The corresponding open-source Python code, previously introduced[8,9], can be found at https://github.com/eldad-a/natural-cubic-smoothing-splines . The smooth factor was calculated independently for each dataset. Two smoothing splines were calculated based on the resulting data: first, the smooth factor was evaluated using the AIC_C, then an over-smoothed spline was evaluated using a 10^{-4} times smaller one. The temporal location of local maxima and minima were identified in the over-smoothed spline. The corresponding intensities were evaluated based on the first spline. A baseline intensity was estimated for each local maximum by linear interpolation of the two neighboring local minima (in case one was outside the dataset, only one was used). The FWHM was defined as the spline intersection with the middle value between the peak and the baseline, in case such intersection existed in the time interval between the neighboring local minima. Finally, a filtering step was applied: peaks were sorted based on their height, evaluated as the difference from the corresponding baseline; starting from the next to highest, each peak height was compared to the previous one; if a ratio smaller than 30% was

found, that peak and all smaller ones were discarded. The results are presented in Supplementary Fig. 5a, b, and in the corresponding figure caption and paragraph in the main text. 19 out of a total of 24 excised meristems were included in the above analysis; the other five out of the 24 samples were excluded as these showed a pattern which was qualitatively distinct from the majority, mostly attributable to experimental interventions, such as water replacement during imaging. Similarly, four out of five intact meristems were included in the analysis. To test whether the measured differences between the two groups, the excised and intact ones, could be associated with the excision of the meristems, we have applied the 2-sided Kolmogorov-Smirnov test (resulting in 0.213, p-value of 0.53, and 0.213, p-value of 0.39 for the inter-peak times and FWHM correspondingly) and Anderson-Darling test for 2-samples (resulting in -0.351, approximated significance level of 0.50, and 0.225, approximated significance level of 0.28, for the inter-peak times and FWHM correspondingly), implemented in SciPy[10]; the differences between the samples are so small that resulting p-values for the Null Hypothesis (no difference) tests are greater than ¼.

To characterize the spikes, for signal frequency, we manually counted the spike event numbers and normalized them based on total cell number and total time during signals were recorded. The total cell number is estimated based on the ratio of the SAM tissue area and the single cell area from 2D images. For individual cell spike duration time, we manually counted the image numbers of individual continuous increased signal from the Ca^{2+} movies with laser scanning intervals of 1s or 2s.

For R-GECO1 fractional fluorescent intensity changes ($(I-I_0)/I_0$) during Ca^{2+} wave propagation induced by mechanical ablation with a glass pipette and direct quantitative comparison between spontaneous Ca^{2+} signal oscillations and Ca^{2+} waves, mean values of fluorescence intensity were extracted from the regions of interest that encompassed the maximum area in the SAM reached by the Ca^{2+} signal using ImageJ. For measuring the effect of dilution series of $LaCl_3$ and BAPTA on Ca^{2+} waves by mechanical perturbation with a glass pipette, mean values of fluorescence intensity were extracted from the regions of interest that encompassed the 2D area in the plane of the SAM (a region that did not include flower primordia) being imaged regardless of the total reached by the Ca^{2+} wave. Fractional ratio changes were calculated from background normalized intensity values as $(I-I_0)/I_0$, where I_0 is the average fluorescence intensity at the resting level. The maximum signal change represents the mean of three individual time frames at (t-1) to (t+1) that cover the maximum peak amplitude.

To compare $LaCl_3$ and BAPTA treatments with water controls in signal area change and wave propagation speed, we extracted these measures from images as follows using MATLAB software: individual frames were segmented to extract the calcium signal region in each frame. First, the signal was treated by a Gaussian smoothing followed by thresholding of the foreground (Supplementary Fig. 9a). Possible artifacts that remain outside of this mask were removed from the mask by selecting only the largest area representing the tissue (Supplementary Fig. 9b). A secondary, variable thresholding step was used to extract the calcium signal region within the masked tissue region (Supplementary Fig. 9c). Once all frames were segmented, a final visual inspection was performed to discard sequences with segmentation errors. Area changes were normalized by calculating $area_t$ / max(area), where max(area) refers to the maximum area found

in any of the three individual frames at (t-1) and (t+1). To quantify the propagation speed of the signal front, we manually marked the wave initiation site on the signal boundary on the first frame of each sequence. The initiation site on the first frame was transferred to subsequent frames by mapping it to the closest point on the boundary. We computed the distance d_t (in microns) from the initiation site to the boundary pixels in five different directions as -60°, -30°, 0°, 30°, 60°, in frame *t* (Supplementary Fig. 9d). The speed of propagation was computed as the average of frame-by-frame distances over consecutive frames, e.g. (d_t – d_{t-1}). Propagation speeds along each direction were averaged over all sequences within each treatment group.

Quantification of MT orientation and PIN1-GFP localization.

MT orientations were quantified using the FibrilTool plugin in ImageJ as published[1]**.** However, since the original lines representing the orientation and anisotropy were hard to visualize, lines easy to see were drawn overlaying the original lines for better visualization. PIN1-GFP signal localization was estimated based on maximum signal intensity localization on the membranes and the behavior of different cells was categorized into 4 groups- (1) maximum PIN1-GFP fluorescence intensity on the membrane next to the wall away from the site of ablation, (2) maximum PIN1-GFP fluorescence intensity on the lateral membranes, (3) uniform PIN1-GFP fluorescence intensity and (4) maximum PIN1-GFP fluorescence intensity on the membrane next to the wall towards the site of ablation. Percentage of cells for each category was counted for both mock-treated and $LaCl_3$ pretreated meristems. Cells located in the radius of up to three cell layers from the site of ablation were included in the analysis.

Supplementary References

1. Boudaoud, A. *et al.* FibrilTool, an ImageJ plug-in to quantify fibrillar structures in raw microscopy images. *Nature Protocols* **9**, 457–463 (2014).
2. Bhatia, N. *et al.* Auxin Acts through MONOPTEROS to Regulate Plant Cell Polarity and Pattern Phyllotaxis. *Curr. Biol.* **26**, 3202–3208 (2016).
3. Heisler, M. G. *et al.* Patterns of Auxin Transport and Gene Expression during Primordium Development Revealed by Live Imaging of the Arabidopsis Inflorescence Meristem. *Current Biology* **15**, 1899–1911 (2005).
4. Keinath, N. F. *et al.* Live Cell Imaging with R-GECO1 Sheds Light on flg22- and Chitin-Induced Transient [Ca2+]cyt Patterns in Arabidopsis. *Molecular Plant* **8**, 1188–1200 (2015).
5. Hamant, O. *et al.* Developmental Patterning by Mechanical Signals in Arabidopsis. *Science* **322**, 1650–1655 (2008).
6. Wasserman, L. *All of Nonparametric Statistics*. (Springer-Verlag, 2006).
7. Hurvich, C. M., Simonoff, J. S. & Tsai, C.-L. Smoothing parameter selection in nonparametric regression using an improved Akaike information criterion. *Journal of the Royal Statistical Society: Series B (Statistical Methodology)* **60**, 271–293 (1998).
8. Afik, E. Robust and highly performant ring detection algorithm for 3d particle tracking using 2d microscope imaging. *Scientific Reports* **5**, 13584 (2015).
9. Afik, E. & Steinberg, V. On the role of initial velocities in pair dispersion in a microfluidic chaotic flow. *Nature Communications* **8**, 468 (2017).
10. Jones, E., Oliphant, T. & Peterson, P. SciPy: Open source scientific tools for Python. (2001).
