# Supplementary material for: Calcium signals are necessary to establish auxin transporter polarity in a plant stem cell niche
Source: Nat Commun. 2019 Feb 13;10:726. doi: 10.1038/s41467-019-08575-6 (PMC6374474; doi:10.1038/s41467-019-08575-6)
Supplement: Supplementary file 3 — Description of Additional Supplementary Files [file 41467_2019_8575_MOESM3_ESM.pdf]

Description of Additional Supplementary Files

File Name: Supplementary Movie 1
Description: movie of 2D color-coded Ca2+ spontaneous oscillation in the SAM expressing R-GECO1 as in Fig. 3a.

File Name: Supplementary Movie 2
Description: movie of 2D color-coded Ca2+ spontaneous oscillation in the SAM that shows different initiation sites over time. Plasma membrane signal was labeled as green color.

File Name: Supplementary Movie 3
Description: movie of 2D color-coded Ca2+ spontaneous oscillation in the SAM from the intact plant expressing R-GECO1.

File Name: Supplementary Movie 4
Description: Spikes predicted by GCaMP6f in the Arabidopsis SAM. Close-up view from above of a confocal projection of the Arabidopsis SAM comprising of 33 Z-stacks showing spikes predicted by GCaMP6f calcium sensor. Each stack comprises of 13 optical slices recorded over a period of 2.126 minutes with a scan rate of 1 stack per 3.2 seconds and 1 optical slice per 0.3 seconds. Scale bar, 20µm.

File Name: Supplementary Movie 5
Description: movie of 2D color-coded Ca2+ spontaneous signals in the SAM expressing R-GECO1 during 0.5M LaCl3 treatment.

File Name: Supplementary Movie 6
Description: movie of 2D color-coded Ca2+ spontaneous signals in the SAM expressing R-GECO1 during 0.2M BAPTA treatment.

File Name: Supplementary Movie 7
Description: movie of Ca2+ wave during mechanical perturbation through a pipette in the same SAM expressing R-GECO1 as in Fig. 3c-f.

File Name: Supplementary Movie 8
Description: Calcium wave response induced upon laser induced cellular ablation in the Arabidopsis SAM. A transverse optical section of the SAM showing a calcium wave predicted by GCaMP6f (green) upon laser induced cellular ablation, originating from the site of ablation in the center of the optical section.

File Name: Supplementary Movie 9
Description: movie of Ca2+ signal responses to non-injurious mechanical perturbations (pipette withdrawal) in the same SAM expressing R-GECO1 as in Supplementary Fig. 11a-d.

File Name: Supplementary Movie 10
Description: movie of Ca2+ signal responses to non-injurious mechanical perturbations (pressing and releasing) in the same SAM expressing R-GECO1 as in Supplementary Fig. 11e-h.

File Name: Supplementary Movie 11
Description: movie of Ca2+ signal recovery at 3 h after mechanical stimulation through a pipette to SAMs pretreated with 5mM LaCl3 for 15min in the same SAM expressing RGECO1 as in Supplementary Fig. 12a-e.

File Name: Supplementary Movie 12
Description: movie of Ca2+ signal recovery at 3 h after mechanical stimulation through a pipette to SAMs pretreated with 2mM BAPTA for 10min in the same SAM expressing R-GECO1 as in Supplementary Fig. 12f-j.
