# Supplementary material for: Calcium signals are necessary to establish auxin transporter polarity in a plant stem cell niche
Source: Nat Commun. 2019 Feb 13;10:726. doi: 10.1038/s41467-019-08575-6 (PMC6374474; doi:10.1038/s41467-019-08575-6)
Supplement: Supplementary file 17 — Reporting Summary [file 41467_2019_8575_MOESM17_ESM.pdf]

nature research

Corresponding author(s): Elliot M. Meyerowitz
Marcus G. Heisler

Last updated by author(s): Dec 24, 2018

Reporting Summary

Nature Research wishes to improve the reproducibility of the work that we publish. This form provides structure for consistency and transparency in reporting. For further information on Nature Research policies, see Authors & Referees and the Editorial Policy Checklist.

Statistics

For all statistical analyses, confirm that the following items are present in the figure legend, table legend, main text, or Methods section.

n/a	Confirmed	
☐	☒	The exact sample size (*n*) for each experimental group/condition, given as a discrete number and unit of measurement
☐	☒	A statement on whether measurements were taken from distinct samples or whether the same sample was measured repeatedly
☐	☒	The statistical test(s) used AND whether they are one- or two-sided<br>*Only common tests should be described solely by name; describe more complex techniques in the Methods section.*
☒	☐	A description of all covariates tested
☒	☐	A description of any assumptions or corrections, such as tests of normality and adjustment for multiple comparisons
☐	☒	A full description of the statistical parameters including central tendency (e.g. means) or other basic estimates (e.g. regression coefficient) AND variation (e.g. standard deviation) or associated estimates of uncertainty (e.g. confidence intervals)
☐	☒	For null hypothesis testing, the test statistic (e.g. *F*, *t*, *r*) with confidence intervals, effect sizes, degrees of freedom and *P* value noted<br>*Give P values as exact values whenever suitable.*
☒	☐	For Bayesian analysis, information on the choice of priors and Markov chain Monte Carlo settings
☒	☐	For hierarchical and complex designs, identification of the appropriate level for tests and full reporting of outcomes
☒	☐	Estimates of effect sizes (e.g. Cohen's *d*, Pearson's *r*), indicating how they were calculated

Our web collection on statistics for biologists contains articles on many of the points above.

Software and code

Policy information about availability of computer code

Data collection	We used standard Zeiss510, ZEN Black and Leica LAS-X imaging system software to obtain images, and we used Fiji-ImageJ and Imaris software for image visualization.
Data analysis	We used Python for generating the programming code for quantitative analysis of the meristem calcium oscillations, we used MATLAB2016 software for quantitative analysis of the meristem calcium waves upon mechanical perturbation, and used FibrilTool plugin in ImageJ for quantifying microtubule orientations.

For manuscripts utilizing custom algorithms or software that are central to the research but not yet described in published literature, software must be made available to editors/reviewers. We strongly encourage code deposition in a community repository (e.g. GitHub). See the Nature Research guidelines for submitting code & software for further information.

Data

Policy information about availability of data

All manuscripts must include a data availability statement. This statement should provide the following information, where applicable:

- Accession codes, unique identifiers, or web links for publicly available datasets
- A list of figures that have associated raw data
- A description of any restrictions on data availability

The corresponding open-source Python code on nature-cubic-smoothing-splines can be found at https://github.com/eldad-a/natural-cubic-smoothing-splines. All other data and code are available from the corresponding author upon request, including all of the microscope time lapse files, the programming code for quantitative analysis of the meristem calcium oscillations and waves upon mechanical perturbation. The source data underlying Supplementary Figs 1a-b, 4c, 5a-f, 7e-j, 8a-b, 10, 11i-l, 12k-l and Supplementary Tables 1 and 2 are provided as a Source Data file.

Field-specific reporting

Please select the one below that is the best fit for your research. If you are not sure, read the appropriate sections before making your selection.

☒ Life sciences ☐ Behavioural & social sciences ☐ Ecological, evolutionary & environmental sciences

For a reference copy of the document with all sections, see nature.com/documents/nr-reporting-summary-flat.pdf

Life sciences study design

All studies must disclose on these points even when the disclosure is negative.

Sample size	Sample size was always larger than three for biological replications. Sample sizes were indicated in each result and every statistical analysis.
Data exclusions	Ca2+ spontaneous oscillation peaks were sorted based on their height, evaluated as the difference from the corresponding baseline; starting from the next to highest, each peak height was compared to the previous one; if a ratio smaller than 30% was found, that peak and all smaller ones were discarded. 12 out of 26 BAPTA-treated SAMs from PIN1-GFP reporter were excluded because of the fluorescent signal intensity weakness and internalization. Five out of 24 excised SAMs and one out of five intact SAMs from R-GECO1 reporter were excluded as they showed an irregular oscillation pattern which was qualitatively distinct from the majority. The data selection process is detailed and explained in the main text in the manuscript, along with a description of the data used and not used and the numbers as given above.
Replication	We did multiple biological replications for all chemical treatments on different fluorescent reporters. All percentages and n numbers are indicated in the manuscript.
Randomization	We randomly took samples for both chemical treatments and mock treatments.
Blinding	Blinding is not applicable to our study. We did a confocal imaging based observation for chemical effects on Ca2+ signals and PIN1-GFP protein polarization.

Reporting for specific materials, systems and methods

We require information from authors about some types of materials, experimental systems and methods used in many studies. Here, indicate whether each material, system or method listed is relevant to your study. If you are not sure if a list item applies to your research, read the appropriate section before selecting a response.

Materials & experimental systems

n/a	Involved in the study
☒	☐ Antibodies
☒	☐ Eukaryotic cell lines
☒	☐ Palaeontology
☒	☐ Animals and other organisms
☒	☐ Human research participants
☒	☐ Clinical data

Methods

n/a	Involved in the study
☒	☐ ChIP-seq
☒	☐ Flow cytometry
☒	☐ MRI-based neuroimaging
